# Whole‐genome single nucleotide polymorphism and mating compatibility studies reveal the presence of distinct species in sub‐Saharan Africa *Bemisia tabaci* whiteflies

**DOI:** 10.1111/1744-7917.12881

**Published:** 2020-11-30

**Authors:** Habibu Mugerwa, Hua‐Ling Wang, Peter Sseruwagi, Susan Seal, John Colvin

**Affiliations:** ^1^ Natural Resources Institute University of Greenwich Central Avenue Chatham Maritime Kent UK; ^2^ Department of Entomology University of Georgia Griffin Georgia USA; ^3^ Institute of Insect Sciences Zhejiang University Hangzhou China; ^4^ Biotechnology Department Mikocheni Agricultural Research Institute Dar es Salaam Tanzania

**Keywords:** *Bemisia tabaci*, phylogenomics, reciprocal crossing, sub‐Saharan Africa species

## Abstract

In sub‐Saharan Africa cassava growing areas, two members of the *Bemisia tabaci* species complex termed sub‐Saharan Africa 1 (SSA1) and SSA2 have been reported as the prevalent whiteflies associated with the spread of viruses that cause cassava mosaic disease (CMD) and cassava brown streak disease (CBSD) pandemics. At the peak of CMD pandemic in the late 1990s, SSA2 was the prevalent whitefly, although its numbers have diminished over the last two decades with the resurgence of SSA1 whiteflies. Three SSA1 subgroups (SG1 to SG3) are the predominant whiteflies in East Africa and vary in distribution and biological properties. Mating compatibility between SSA1 subgroups and SSA2 whiteflies was reported as the possible driver for the resurgence of SSA1 whiteflies. In this study, a combination of both phylogenomic methods and reciprocal crossing experiments were applied to determine species status of SSA1 subgroups and SSA2 whitefly populations. Phylogenomic analyses conducted with 26 548 205 bp whole genome single nucleotide polymorphisms (SNPs) and the full mitogenomes clustered SSA1 subgroups together and separate from SSA2 species. Mating incompatibility between SSA1 subgroups and SSA2 further demonstrated their distinctiveness from each other. Phylogenomic analyses conducted with SNPs and mitogenomes also revealed different genetic relationships among SSA1 subgroups. The former clustered SSA1‐SG1 and SSA1‐SG2 together but separate from SSA1‐SG3, while the latter clustered SSA1‐SG2 and SSA1‐SG3 together but separate from SSA1‐SG1. Mating compatibility was observed between SSA1‐SG1 and SSA1‐SG2, while incompatibility occurred between SSA1‐SG1 and SSA1‐SG3, and SSA1‐SG2 and SSA1‐SG3. Mating results among SSA1 subgroups were coherent with phylogenomics results based on SNPs but not the full mitogenomes. Furthermore, this study revealed that the secondary endosymbiont—*Wolbachia*—did not mediate reproductive success in the crossing assays carried out. Overall, using genome wide SNPs together with reciprocal crossings assays, this study established accurate genetic relationships among cassava‐colonizing populations, illustrating that SSA1 and SSA2 are distinct species while at least two species occur within SSA1 species.

## Graphical abstract

Graphical abstract: Cassava colonizing *Bemisia tabaci* whiteflies in sub‐Saharan Africa (SSA) vector viruses causing cassava mosaic and cassava brown streak diseases, responsible for devastating yield losses in cassava—a security food crop in SSA. The species status of these whiteflies is a controversial subject. Using phylogenomic analyses (26 548 205 bp whole genome single nucleotide polymorphisms and full mitogenomes) together with reciprocal crossing assays, the species status of cassava colonizing whitefly populations was resolved.

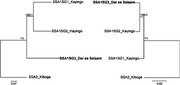

## Introduction

*Bemisia tabaci* species are phloem‐feeding insects that cause considerable damage to a wide range of crops globally (Legg *et al*., 2002; Dinsdale *et al*., 2010). *B*. *tabaci* is a species complex for which more than 40 morphologically indistinguishable, but genetically diverse species have been proposed (Mugerwa *et al*., 2018). Members of the species complex differ greatly in host‐plant range (Zang *et al*., 2006; Xu *et al*., 2011), inducement of phytotoxic disorders (Sseruwagi *et al*., 2005; Vyskočilová *et al*., 2018), resistance to insecticides (Wang *et al*., 2012; Horowitz & Ishaaya, 2014), invasiveness (Brown *et al*., 1995; Liu *et al*., 2007), and specificity of begomovirus transmission (Fiallo‐Olivé *et al*., 2020), which creates challenges in identifying and managing them. The most significant threat posed by *B*. *tabaci* is the ability of its members collectively to vector over 300 plant viruses belonging predominantly to the genus *Begomovirus*, but also including devastating plant viruses from the genera *Carlavirus*,*Crinivirus*, *Ipomovirus*, *Polerovirus*, and *Torradovirus* (Ng & Falk, 2006; Amari *et al*., 2008; Navas‐Castillo *et al*., 2011; Zanardo & Carvalho, 2017; Ghosh *et al*., 2019). In sub‐Saharan Africa (SSA), *B*. *tabaci* vectors cassava mosaic begomoviruses (CMBs) (Storey & Nichols, 1938) and cassava brown streak ipomoviruses (CBSIs) (Maruthi *et al*., 2005; Mware *et al*., 2009). Annual economic losses attributed to cassava mosaic disease (CMD) and cassava brown streak disease (CBSD) in cassava are estimated at US$1.9–2.7 billion and US$0.1 billion, respectively (Legg *et al*., 2006; Ndunguru *et al*., 2015). The spread of both CMD and CBSD viruses has been correlated with high whitefly populations often referred to as “superabundant” and a combination of factors have been attributed to the generation of these high whitefly populations (Legg *et al*., 2002; Colvin *et al*., 2006; Omongo *et al*., 2012; Macfadyen *et al*., 2018; Mugerwa *et al*., 2019). Whether these high whitefly populations represent the same species remains a matter of controversy (Legg *et al*., 2014; Wosula *et al*., 2017).

To date, the most popular and widely used molecular marker to resolve the classification of *B. tabaci* species has been the partial (657 bp) mitochondrial cytochrome oxidase 1 (*mtCO1*) sequences at the 3’ end barcode region; *mtCO1* sequence divergence of ≥3.5%–4.0% are designated as different *B. tabaci* species (Dinsdale *et al*., 2010; Lee *et al*., 2013; Boykin *et al*., 2018; Mugerwa *et al*., 2018). Although the *mtCO1* marker has been used widely to delineate species boundaries in *B*. *tabaci*, it has several drawbacks and some of these include: (i) the maternally inherited characteristic make it unable to identify hybrid progeny (Birky, 2001; Vyskočilová *et al*., 2018); and (ii) the invasion and spread of maternally inherited secondary endosymbionts through arthropods can drive a single mitochondrial haplotype through a population, so that insects with divergent nuclear genes can have uniform mitochondrial genes (Galtier *et al*., 2009). In addition, inaccurate determination of genetic differences and population histories among samples (Hadjistylli *et al*., 2016; Wosula *et al*., 2017; Vyskočilová *et al*., 2018) was reported whilst using mitochondrial DNA. For example, the Mediterranean species (MED) was formerly recognized to possess four subgroups (SG) namely Q1, Q2, Q3 and African silver leafing (ASL) based on the partial *mtCO1* sequences (Gueguen *et al*., 2010), but Vyskočilová *et al*. (2018) demonstrated the existence of at least two distinct species within MED species “subgroups” based on mating compatibility.

Using the partial *mtCO1* marker, five distinct members of *B*. *tabaci*, namely SSA1‐5, have been reported to colonizing cassava in sub‐Saharan Africa (SSA) (Legg *et al*., 2002; Berry *et al*., 2004). Within SSA1 species, five subgroups designated as SG1 to SG5 based on >0.6% divergence in partial *mtCO1* sequences have also been reported (Mugerwa *et al*., 2012; Legg *et al*., 2014). Among the identified SSA1 subgroups, only SG1 to SG3 are the prevalent whiteflies in East and Central Africa (ECA) (Wosula *et al*., 2017; Ally *et al*., 2019). Various studies (Sseruwagi *et al*., 2005; Mugerwa *et al*., 2012; Legg *et al*., 2014; Ghosh *et al*., 2015; Ally *et al*., 2019) have shown that SSA1‐SG1 gradually became the predominant whitefly on cassava and extended its geographical range within ECA, whilst displacing SSA2 whiteflies from early 2000s to the present. The displacement of SSA2 whitefly by whiteflies of SSA1 subgroups was attributed to the possible interbreeding between these two whiteflies, as reported by Maruthi *et al*. (2004), resulting in genotypes with SSA1 subgroups mitochondrial genomes, but with fecundity traits of SSA2 (Mugerwa *et al*., 2012). Whether or not these two species (SSA1 subgroups and SSA2) can be classified as distinct species, since their partial *mtCO1* sequences diverge by >8.0 nt, is a subject still worth further investigation, by using a combination of different approaches including full mitogenomes analysis, nuclear genome analyses and mating studies. In addition, SSA1 subgroups (SG1 to SG3) although currently identified as the same species, differ in their field abundance and distribution (Legg *et al*., 2014; Ghosh *et al*., 2015), host range and adaptation (Mugerwa *et al*., 2018), and fecundity and survivorship (Mugerwa *et al*., 2019). These observed biological and ecological traits among SSA1 subgroups are suggestive of distinct species within SSA1, however, this hypothesis has yet to be proven using the methods/tools stated above.

Next‐generation sequencing (NGS) technology, with its high‐throughput capacity and low cost, has become an important analytical tool for species identification (Yang *et al*., 2014). Using this technology, draft genomes of the Mediterranean (MED), Middle‐East Asia Minor 1 (MEAM1) and Sub‐Saharan Africa—East and Central Africa (SSA‐ECA) species have been generated (Chen *et al*., 2016, 2019; Xie *et al*., 2017). It should be noted that SSA‐ECA is a naming system proposed by Wosula *et al*. (2017) to refer to SSA1‐SG1 species. NGS can generate dense genomic markers for re‐analysis of species relationships in an unprecedented way. However, solely based on genomic datasets, determination of species status will raise debates as presently observed for cassava‐colonising whiteflies. For example, Wosula *et al*. (2017) used 7453 single nucleotide polymorphisms (SNPs) distributed across the whole genome to delineate African cassava colonizing whitefly populations into six distinct major groups. Reanalysis of the Wosula *et al*. (2017) dataset by Elfekih *et al*. (2019) revealed that the naming proposed by the former was unnecessary since the effects of pseudogenes in the partial *mtCO1* sequences had not been considered. Further, Elfekih *et al*. (2019) reported a lack of evidence to support the presence of five subgroups in SSA1 species, albeit that two subspecies were clearly present within this species. Thus, to avoid the above limitations and to identify the cryptic species within cassava whitefly populations correctly, a combination of mating bioassays and phylogenomics was adopted in this study.

To test the two hypotheses, (i) SSA1 whiteflies are distinct species from SSA2 whiteflies as supported by >8.0% nt sequence divergence in their partial *mtCO1* sequences and (ii) distinct species exist in SSA1 whiteflies based on ecological and biological data for the different subgroups, we performed genome resequencing of adult whiteflies collected from four laboratory colonies of cassava whiteflies (SSA1‐SG1, SSA1‐SG2, SSA1‐SG3, and SSA2) established from cassava field collections. In addition, reciprocal mating assays were carried out under laboratory conditions between the four colonies. The endosymbiont status of the four colonies was determined as this can affect mating compatibility (Yen & Barr, 1971; Binnington & Hoffmann, 1989; Werren, 1997). Finally, the molecular relationships of individuals collected from the four whitefly colonies based on assembled full mitogenomes and whole genomic homozygous SNPs were compared to results obtained from the reciprocal crossing assays to provide proof of existence of biological species in cassava whitefly populations.

## Materials and methods

### Establishment of cassava whitefly colonies

Approximately 100–200 adult *B. tabaci* whiteflies were collected from cassava fields in Kayingo and Kiboga in Uganda and the Dar es Salaam area in Tanzania in 2016 and 2013, respectively, using an aspirator and transferred to fresh cassava shoots in “Lock & Lock” (LL) containers (Wang *et al*., 2011). The stem of each cassava shoot was dipped in water in the lower part of the LL container to prevent the shoots from wilting whilst in transit. Upon reaching the Natural Resources Institute (NRI) at the University of Greenwich in UK, the surviving *B. tabaci* whiteflies were transferred to LL containers with 20‐d‐old aubergine plants to initiate the “field”‐collected colonies of unknown and mixed identities (based on *mtCO1* marker). Ten pairs (female and male) of whiteflies were collected from each of the three field‐collected colonies to setup isofemale lines (10 × 3 = 30 isofemale lines). Upon establishment, their identity was determined using the partial *mtCO1* marker as described in the following paragraphs. Based on the reproductive vigour of the established isofemale line and its *mtCO1* identity, four isofemale lines representing SSA1‐SG1, SSA1‐SG2, SSA1‐SG3, and SSA2 species were selected for mating studies.

Established isofemale whitefly colonies initially set up in LL containers were transferred to BugDorm insect‐rearing rectangular cages (47.5 × 47.5 × 93 cm, MegaView Science Co., Ltd., Taiwan, China) and maintained at 28 ± 2°C, 30% r.h. and 14 : 10 L : D. The four isofemale whitefly colonies used in the mating experiment were: SSA1‐SG1_Kayingo and SSA1‐SG2_Kayingo, SSA1‐SG3_Dar es Salaam and SSA2_Kiboga established from whiteflies collected from Kayingo (Uganda), Dar es Salaam (Tanzania) and Kiboga (Uganda), respectively. Kayingo and Kiboga had high whitefly populations (>200 whiteflies for the top five leaves) compared with Dar es Salaam, which had low whitefly populations (<20 whiteflies for the top five leaves) (unpublished data).

### DNA extraction from an individual whitefly

Genomic DNA was extracted from individual whiteflies collected from the four established pure colonies using the DNeasy animal tissue kit (Qiagen, Germany) following the manufacturer's protocol. Obtained DNA from either male or female whiteflies was used for PCR amplifications followed by Sanger sequencing. For the purpose of Illumina sequencing, genomic DNA was extracted from only individual *B. tabaci* females collected from SSA1‐SG1_Kayingo, SSA1‐SG2_Kayingo, SSA1‐SG3_Dar es Salaam, and SSA2_Kiboga colonies.

### Amplification, gel electrophoresis, and purification of PCR products

PCR reaction mixtures (20 *μ*L) were set up containing 10 *μ*L of 2× reSource™ Taq Mix (reSource Taq DNA Polymerase, 6 mmol/L MgCl_2_, 2 mmol/L dNTPs, enhancers, and stabilizers) (Source BioScience, Nottingham, UK), 0.5 μmol/L of forward and reverse primer, 6 *μ*L of molecular grade water and 2 *μ*L of DNA template. A negative control (molecular biology grade water used in the place of DNA template) was included in every PCR run. PCR cycling was performed in a 2720 Thermal cycler (Applied Biosystems, UK) programmed as follows: initial denaturation at 94°C for 2 min, 35 cycles of 94°C for 30 s, annealing at different temperatures depending on primer set for 30 s (Table S1), and primer extension at 72°C for 1 min, with a final 10 min elongation cycle at 72°C. To examine the endosymbionts infection in the four established pure colonies, the commonly used 16S RNA markers were adopted as described by Jiang *et al*. (2012). For the primary endosymbiont bacteria screening, no positive control was included in the screening because the presence of the endosymbiont in the whitefly is obligatory (Jiang *et al*., 2012). As for each secondary endosymbiont, various species known to contain the related secondary endosymbionts were included in the screening. DNA extracted from SSA1‐SG3, Asia II‐1, Israel MEAM1, Asia I, and New World *B. tabaci* were used as positive controls for *Arsenophonus*, *Cardinium*, both *Hamiltonella* and *Rickettsia*, *Wolbachia* and *Fritschea*, respectively (Vyskočilová *et al*., 2018).

PCR products were visualized through agarose gel electrophoresis and amplified PCR products of the expected base pair sizes were purified using reSource™ PCR purification kit as per the manufacturer's instructions (Source BioScience, UK).

### Restriction of PCR products, Sanger sequencing and analysis

Purified *mtCO1* PCR products were set up for restriction digestion as described by Ghosh *et al*. (2015). Restriction digestion reaction mixtures (20 *μ*L) were set up and contained 2 *μ*L 10× NEBuffer, 0.2 *μ*L Apo I (10 U/*μ*L), 3.8 μL molecular grade water, and 14 *μ*L purified PCR product. The digestion mixture was mixed by pipetting 4–5 times and incubated at 50°C for 1 h. The digested products were visualized under UV light after agarose gel electrophoresis. Expected sizes of restricted PCR *mtCO1* products for SSA1‐SG1 individuals were 654, 122, and 91 bp while for SSA1‐SG2 individuals were 493, 252, and 122 bp.

Purified PCR products were sent for Sanger sequencing at Source Bioscience (Nottingham, UK). Sequences obtained from purified PCR products were edited manually for each individual sequence using the *MEGA* version 6 software program (Tamura *et al*., 2013). Edited sequences were used for similarity searches in the National Centre for Biotechnology Information (NCBI) GenBank databases (www.ncbi.nlm.nih.gov).

### Library construction and Illumina Sequencing

The Illumina pair‐end sequencing (PE150) platform was selected for genome resequencing. All genomic DNA extracted from individual *B. tabaci* used for sequencing library construction with insert sizes of ∼300 bp were prepared following Illumina's standard genomic DNA library preparation procedure. Briefly, purified genomic DNA was sheared into smaller fragments with a desired size by Covaris, and T4 DNA polymerase was applied to generate blunt ends. After adding an “A” base to the 3' end of the blunt phosphorylated DNA fragments, adapters were ligated to the ends of the DNA fragments. The desired fragments were purified through gel‐electrophoresis, then selectively enriched and amplified by PCR. The index tag was introduced into the adapter at the PCR stage as appropriate followed by a library quality test. Finally, the quantified Illumina pair‐end library were used for Illumina HiSeq sequencing (150 bp *2).

### Variation detection and annotation

Raw sequencing data was generated by Illumina base calling software CASAVA v1.8.2 (http://support.illumina.com/sequencing/sequencing_software/casava.ilmn) according to its corresponding guidance. Illumina sequence data were first examined using FastQC v.0.10.1 (http://www.bioinformatics.babraham.ac.uk/projects/fastqc/) to check for irregularities or sequencing errors. The raw paired‐end reads were trimmed and quality controlled by Trimmomatic v0.33 (http://www.usadellab.org/cms/uploads/supplementary/Trimmomatic) (Bolger *et al*., 2014). The high‐quality sequencing reads were aligned to the SSA‐ECA genome sequence obtained from http://www.whiteflygenomics.org/cgi-bin/bta/index.cgi using BWA‐MEM v 0.7.12‐r1039 (http://bio-bwa.sourceforge.net/) software (Li, 2013). After removing PCR‐duplication reads by SAMtools v0.1.18 (r982:295) (http://samtools.sourceforge.net/), the sequencing depth and coverage were calculated based on the alignments by custom perl scripts. The valid BAM file were used to detect SNPs by GATK v2.7‐2 “UnifiedGenotyper” function (http://www.broadinstitute.org/gatk/) with the parameters of “‐stand_call_conf 50 ‐stand_emit_conf 50 ‐dcov 2000.” The annotations of detected variations were performed by ANNOVAR (http://www.openbioinformatics.org/annovar/), including SNP (synonymous or non‐synonymous mutations of SNPs) (Wang *et al*., 2010).

### Phylogenetic analysis

Thirteen protein coding genes (PCG) and the two ribosomal RNA (16S and 12S) from four assembled mitogenomes were aligned by translation alignment with genetic code of Invertebrate Mitochondrial and MUSCLE Alignment, respectively. All 15 genes used in these alignments were concatenated and partitioned by AMAS (Borowiec, 2016). PartitionFinder 2 (Lanfear *et al*., 2016) was used for selecting partitioned models of mitochondrial evolution for phylogenetic analyses. The input partitions include nucleotide sequences of 13 PCGs together with *16S* and *12S*, as well as the position of each gene and its relating codon. The output minimized the input partitions as seven subsets as follows: (i) *12S*, *16S*; (ii) *cytb*, *atp6*; (iii) *nad6*, *nad2*, *atp8*; (iv) *cox1*, *cox2*; (v) *nad3*, *cox3*; (vi) *nad1*, *nad4*, *nad4l*; (vii) *nad5*. Regarding the models chosen by PartitionFinder, two models were selected for these seven parts: GTR model for the subset of 16S and 12S, and GTR+G model for the remaining subsets. The phylogenetic trees were constructed using IQ‐TREE v1.5.5 (Nguyen *et al*., 2015) with 1000 rapid bootstraps replicates and a Bayesian method (MrBayes v. 3.2; Ronquist & Huelsenbeck, 2003) according to the mixed models generated from PartitionFinder 2 (Lanfear *et al*., 2016). Phylogenetic analyses using homozygous SNPs (26 548 205 bp) were performed with IQ‐TREE v1.5.5 (Nguyen *et al*., 2015). The best nucleotide substitution model was chosen by model test function in IQ‐TREE. Finally, the transversion plus empirical base frequencies model (TVM+F) was used for phylogeny inference of homozygous SNPs. The program was run with 1000 bootstrap replicates. We also used RAxML (Stamatakis, 2014) to perform relatedness analysis using identity, which generated consistent tree topologies.

### Crossing experiments

Reciprocal crosses (SSA1‐SG1 × SSA1‐SG2; SSA1‐SG1 × SSA1‐SG3; SSA1‐SG1 × SSA2; SSA1‐SG2 × SSA1‐SG3; SSA1‐SG2 × SSA2; SSA1‐SG3 × SSA2) were set up between four populations. Control or intrapopulation crosses consisted of a virgin female and males from the same population. This resulted in 12 interpopulation crosses and four control crosses. Interpopulation crosses were replicated 7–10 times, while control crosses were replicated 3–9 times.

The experimental procedure involved trimming a 20‐d‐old aubergine plant to remain with a single leaf before transferring it to a LL container. A single virgin female (♀) and three virgin male (♂) adults in glass tubes (5 × 0.5 cm) were introduced to the aubergine plant in a LL container whose top was covered with tissue paper. At 2‐d intervals, leaves were inspected for the introduced whiteflies. In cases where the males died, they were replaced with live ones.

After 7 d of feeding, mating and the female ovipositing, all the whiteflies were removed from the aubergine plant in the LL container and stored in 90% ethanol. The laid eggs were observed for development until the fourth instar growth stage. After day 20 of the experiment, the plants were inspected daily for emerged adults. The number of emerged whitefly offspring were counted and recorded by sex (male or female) and stored in 90% ethanol. In the reciprocal interpopulation crosses where female offspring emerged, their fertility was examined through carrying out crosses with their male siblings.

Statistical analyses were performed using R (R Development Core Team, 2011; www.R-project.org). Emerged progeny counts were analysed using a negative binomial generalised linear model with log link in the MASS package (Venables & Ripley, 2002). The percentage of females in emerged progeny were analysed using a quasibinomial generalised linear model with binomial errors and logit transformation in the MASS package. A Tukey's HSD test was used for multiple comparisons using multcomp package (Hothorn *et al*., 2008) to determine significant differences between progeny counts and proportion of females in the emerged progeny. Multiple comparisons were displayed by compact letter display using the cld function.

## Results

### Comparison between phylogenomic topology of mitochondrial and whole genomic homozygous SNPs

To infer the molecular phylogenetic relationships among the individual *B. tabaci* populations (SSA1‐SG1, SSA1‐SG2, SSA1‐SG3, and SSA2), phylogenomic trees were constructed using the full mitogenomes and genomic scale biallelic SNPs. The full length of mitogenomes of SSA1‐SG1, SSA1‐SG2, SSA1‐SG3, and SSA2 specimens were 15 220 bp, 15 225 bp, 15 232 bp, and 15 246 bp, respectively. Gene orders were the same as in published mitogenomes of *B*. *tabaci* (Wang *et al*., 2013; Tay *et al*., 2016). Based on the 657 bp partial *mtCO1* sequences, SSA1 subgroups are similar to each other and SSA2 species by 98.48%–98.78% and 91.32%–92.09%, respectively (Table 1). For the genomic datasets, the mapping parameters of the four whitefly females are listed in Table 2 and indicate that sequencing was of high quality and coverage. The mapping rate was high, ranging from 92%–96%, which was expected due to the close relationships of the SSA populations used to the reference SSA‐ECA species (Chen *et al*., 2019).

**Table 1:** Partial mitochondrial cytochrome oxidase 1 gene (657 bp) sequence identities between samples that were subjected for both single nucleotide polymorphism and mitogenome sequencing

	SSA2	SSA1‐SG1	SSA1‐SG3	SSA1‐SG2
SSA2	–			
SSA1‐SG1	91.78%	–		
SSA1‐SG3	92.09%	98.78%	–	
SSA1‐SG2	91.32%	98.48%	98.48%	–

**Table 2:** Mapping parameters to the Sub‐Saharan Africa—East and Central Africa (SSA‐ECA) *Bemisia tabaci* genome

Sample	Clean	Mapped‐reads	Mapped‐rate (%)	Coverage (%)	Aver‐depth
SSA2	193 949 796	185 144 475	0.95	0.90	45.95
SSA1‐SG1	242 663 488	222 983 479	0.92	0.92	56.02
SSA1‐SG2	203 532 908	192 399 657	0.95	0.91	48.71
SSA1‐SG3	193 674 852	185 327 465	0.96	0.90	46.10

**Fig. 1:**
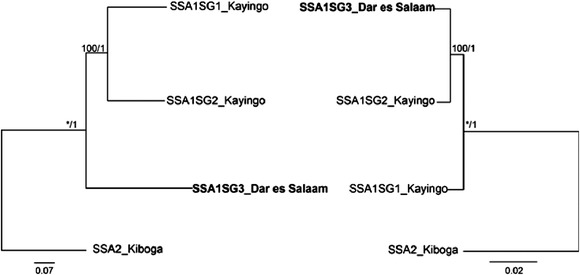
Maximum‐likelihood trees of the *Bemisia tabaci* samples used in th study. Nuclear biallelic SNPs (left, 12 million bp) and mitogenome (right, 12 868 bp) trees constructed by IQ‐TREE from genomic scale biallelic SNPs and mitochondrial genomes. Bootstrap of ML tree and prior of Bayesian tree is shown on nodes.

The phylogenetic analyses were conducted with concatenation of 13 protein coding genes (PCGs) and 16S rRNA (12 868 bp) from mitogenomes and whole genomic homozygous SNPs (26 548 205 bp). The nuclear tree topology generated from whole genomic homozygous SNPs was different from the mitochondrial tree, which was not affected by partition schemes and inference methods (Fig. 1). The tree based on whole genomic SNPs (nuclear) clustered SSA1‐SG1 and SSA1‐SG2 together and separate from the SSA1‐SG3, while the mitochondrial tree clustered SSA1‐SG2 and SSA1‐SG3 together and separated from the SSA1‐SG1 sample. All trees generated from mitogenomes or whole genomic SNPs indicated that all the SSA1 subgroup samples were closer to each other than to the SSA2 sample.

### Crossing experiments

All the populations used for draft single whitefly genome sequencing were subjected to crossing experiments. Progeny were produced in all of the 12 interpopulation and four intrapopulation crosses. However, female progeny were produced in only intrapopulation crosses and two interpopulation crosses (Table 3). The percentage of female progeny produced in the interpopulation cross 1♀ SSA1‐SG1 × 3♂ SSA1‐SG2 (59.1% ± 6.0%) wase lower but not significantly (*P* = 0.072) different to those produced in its respective intrapopulation cross 1♀ SSA1‐SG1 × 3♂ SSA1‐SG1 (80.9% ± 3.6%) (Table 3). Likewise, the percentage of female progeny produced in the interpopulation cross 1♀ SSA1‐SG2 × 3♂ SSA1‐SG1 (32.4% ± 3.5%) was lower but not significantly (*P* = 0.074) different to those produced in its respective intrapopulation cross 1♀ SSA1‐SG2 × 3♂ SSA1‐SG2 (53.1% ± 5.5%) (Table 3).

**Table 3:** Mean ± SE number of progeny and percentage of females in the progeny of reciprocal crosses between four *Bemisia tabaci* cassava populations. The *B. tabaci* used in these crosses were collected from Kayingo (KAY, Uganda), Kiboga (KIB, Uganda), and Dar es Salaam (DAR, Tanzania)

Crosses1♀ × 3♂		No. of replicates	Mean no. of progeny ± SE	% Female progeny
Interpopulation crosses				
1.	SSA1‐SG1 (KAY) × SSA1‐SG2 (KAY)	7	18.9 ± 3.8 a	59.1 ± 6.0 ac
2.	SSA1‐SG2 (KAY) × SSA1‐SG1 (KAY)	10	34.9 ± 5.6 ab	32.4 ± 3.5 b
3.	SSA1‐SG1 (KAY) × SSA1‐SG3 (DAR)	8	30.4 ± 5.5 ab	0.0 ± 0.0
4.	SSA1‐SG3 (DAR) × SSA1‐SG1 (KAY)	9	37.3 ± 6.3 ab	0.0 ± 0.0
5.	SSA1‐SG1 (KAY) × SSA2 (KIB)	8	31.6 ± 5.7 ab	0.0 ± 0.0
6.	SSA2 (KIB) × SSA1‐SG1 (KAY)	10	44.7 ± 7.1 ab	0.0 ± 0.0
7.	SSA1‐SG2 (KAY) × SSA1‐SG3 (DAR)	9	43.1 ± 7.3 ab	0.0 ± 0.0
8.	SSA1‐SG3 (KIB) × SSA1‐SG2 (KAY)	8	26.1 ± 4.8 ab	0.0 ± 0.0
9.	SSA1‐SG2 (KAY) × SSA2 (KIB)	7	50.1 ± 9.5 b	0.0 ± 0.0
10.	SSA2 (KIB) × SSA1‐SG2 (KAY)	8	46.0 ± 8.2 ab	0.0 ± 0.0
11.	SSA1‐SG3 (DAR) × SSA2 (KIB)	9	42.7 ± 7.2 ab	0.0 ± 0.0
12.	SSA2 (KIB) × SSA1‐SG3 (DAR)	10	45.4 ± 7.2 ab	0.0 ± 0.0
Intrapopulation crosses				
13.	SSA1‐SG1 (KAY) × SSA1‐SG1 (KAY)	5	46.2 ± 10.4 ab	80.9 ± 3.6 a
14.	SSA1‐SG2 (KAY) × SSA1‐SG2 (KAY)	3	54.0 ± 15.6 ab	53.1 ± 5.5 bc
15.	SSA1‐SG3 (DAR) × SSA1‐SG3 (DAR)	9	38.1 ± 6.5 ab	36.2 ± 3.6 bc
16.	SSA2 (KIB) × SSA2 (KIB)	9	43.6 ± 7.3 ab	48.5 ± 3.5 bc

Means and percentage progeny followed by the different letters differ significantly at *P* < 0.05.

The number of progeny produced in intrapopulation crosses 1♀ SSA1‐SG1 × 3♂ SSA1‐SG1 (46.2 ± 10.4) and 1♀ SSA1‐SG2 × 3♂ SSA1‐SG2 (54.0 ± 15.6) were high but not significantly (*P* > 0.05) different to those produced in their respective interpopulation crosses 1♀ SSA1‐SG1 × 3♂ SSA1‐SG2; 1♀ SSA1‐SG1 × 3♂ SSA1‐SG3; 1♀ SSA1‐SG1 × 3♂ SSA2 (18.9±3.8‒34.9±5.6) and 1♀ SSA1‐SG2 × 3♂ SSA1‐SG1; 1♀ SSA1‐SG2 × 3♂ SSA1‐SG3; 1♀ SSA1‐SG2 × 3♂ SSA2 (34.9 ± 5.6–50.1 ± 9.6) (Table 3). In contrast, the number of progeny produced in the intrapopulation cross 1♀ SSA2 × 3♂ SSA2 (43.6 ± 7.3) were low but not significantly (*P* > 0.05) different to those produced in its respective interpopulation crosses 1♀ SSA2 × 3♂ SSA1‐SG1; 1♀ SSA2 × 3♂ SSA1‐SG2; 1♀ SSA2 × 3♂ SSA1‐SG3 (44.7 ± 7.1–46.0 ± 8.2) (Table 3). There was no specific trend observed in the number of progeny produced in intrapopulation cross 1♀ SSA1‐SG3 × 3♂ SSA1‐SG3 (38.1 ± 6.5) and its respective interpopulation crosses 1♀ SSA1‐SG3 × 3♂ SSA1‐SG1; 1♀ SSA1‐SG3 × 3♂ SSA1‐SG2; 1♀ SSA1‐SG3 × 3♂ SSA2 (26.1 ± 4.8–42.7 ± 7.2) (Table 3).

The pooled number of progeny produced in both intra‐ and interpopulation crosses were significantly (*P* = 0.025, GLM analysis of deviance) affected by the identity of their female parent (Table S2). The pooled number of progeny produced by SSA2 female parents (44.9 ± 3.9) were significantly different from the pooled number of progeny produced by SSA1‐SG1 female parents (30.7 ± 3.2). The pooled number of progeny produced in both intra‐ and interpopulation crosses were not significantly (*P* = 0.468, GLM analysis of deviance) affected by the identity of their male parents.

The fertility of F1 female progeny produced in interpopulation crosses was confirmed through carrying out reciprocal crossings with their male siblings. F1 female progeny generated from cross 1♀ SSA1‐SG1 × 3♂ SSA1‐SG2 produced 48.0% ± 11.3% F2 female progeny while F1 female progeny produced from cross 1♀ SSA1‐SG2 × 3♂ SSA1‐SG1 produced 25.9% ± 14.3% F2 female progeny (Table S3).

### Endosymbiont status of whitefly colonies

As expected, partial 16S rDNA sequences of the primary endosymbiont *Portiera aleyrodidarum* were PCR‐amplified from all *B. tabaci* individuals examined (Table S4). Six secondary endosymbionts were screened for in the four established pure colonies at NRI and only *Wolbachia* was detected in one colony. Partial 16S rDNA sequences of *Wolbachia* were PCR‐amplified from individual whiteflies collected from a SSA1‐SG2 *B. tabaci* colony established from Kayingo (Table S4). The partial 16S rDNA nucleotide sequence of *Wolbachia* detected in SSA1‐SG2 *B. tabaci* individual was 99.2% and 100% identical to partial 16S rDNA sequences of *Wolbachia* detected in *Drosophila mauritiana* and *Culex pipens*, respectively (Table S5).

### Molecular identity and endosymbionts status of hybrids

**Fig. 2:**
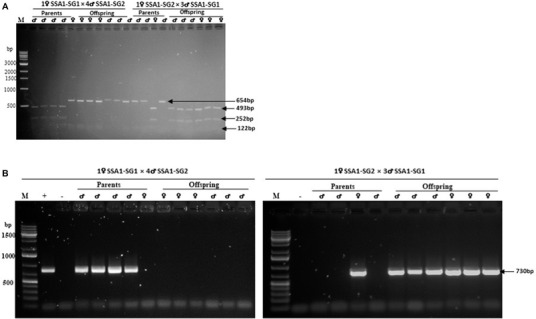
Restriction patterns of the digested partial *mtCO1* gene PCR product and detection of partial 16S rDNA sequences of *Wolbachia* in parents used in reciprocal crosses and offspring generated. (A) Different restriction patterns of the digested partial *mtCO1* PCR product as described by Ghosh *et al*. (2015) used to determine the identity of parents used in reciprocal crosses and offspring generated. Lane M is 1 kb DNA ladder. Restriction pattern of female parent is the same as her offspring. (B) PCR detection of partial 16S rDNA sequences of *Wolbachia* in parents used in reciprocal crosses and offspring generated. Lane M is 2‐Log DNA ladder, lane “+” is positive control, DNA extracted from Asia I *Bemisia tabaci*, and lane “–” is negative control, molecular grade water (Sigma‐Aldrich, UK) used in the place of DNA template. Symbols ♀ and ♂ represent female and male whiteflies, respectively.

In interpopulation crosses 1♀ SSA1‐SG1 × 3♂ SSA1‐SG2 and 1♀ SSA1‐SG2 × 3♂ SSA1‐SG1 where female progeny were produced, the *mtCO1* identity of one set of parents used in the crosses were checked against the *mtCO1* identity of their respective progeny (three females and three males). The observed restriction pattern of the SSA1‐SG1 female parent and her progeny was 654 bp and 122 bp while that of the SSA1‐SG2 female parent and her progeny was 493 bp, 252 bp, and 122 bp. The restriction patterns of both female and male offspring were confirmed as being the same as their female parent and different from that of their male parents (Fig. 2A).

In addition to determining the molecular identity of parents used in the above interpopulation crosses, the secondary endosymbiont: *Wolbachia* was screened in both the parents and progeny generated. Partial 16S rDNA sequences of *Wolbachia* were detected by PCR in all SSA1‐SG2 parents used for the two interpopulation reciprocal crosses; however, they were not amplified from the SSA1‐SG1 parents (Fig. 2B). All the selected progeny produced in cross 1♀ SSA1‐SG2 × 3♂ SSA1‐SG1 were PCR positive for 16S rDNA sequences of *Wolbachia*; however, negative results were produced in the selected progeny produced in cross 1♀ SSA1‐SG1 × 4♂ SSA1‐SG2. The presence or absence of the partial 16S rDNA sequences of *Wolbachia* in progeny produced in the two interpopulation crosses 1♀ SSA1‐SG2 × 4♂ SSA1‐SG1 and 1♀ SSA1‐SG1 × 3♂ SSA1‐SG2, respectively, was dependent on the infection status of their female rather than male parents since this secondary endosymbiont is maternally inherited (Werren, 1997). The presence and absence of partial 16S rDNA sequences of *Wolbachia* in the parents, SSA1‐SG2 and SSA1‐SG1 *B. tabaci* populations, respectively, used in the two interpopulation crosses, acted as additional confirmation of the identity of parents used in these crosses.

## Discussion

Using a genome resequencing approach and reciprocal crossing experiments, our study confirmed our first hypothesis: “SSA1 whiteflies are distinct species from SSA2 whiteflies.” Phylogenomic relationships revealed through the use of both 26 548 205 whole genome SNPs and full mitogenomes (15 220–15 246 bp) clustered SSA1 subgroups together, but separate from SSA2 whiteflies, confirming that SSA1 and SSA2 whiteflies are distinct from one another. In addition, the production of only male progeny in reciprocal crosses carried out between three SSA1 subgroup populations and SSA2 *B. tabaci* species demonstrated mating incompatibility between SSA1 and SSA2 populations. Our results are in agreement with Wosula *et al*. (2017), who reported that there was no gene flow between the SSA1 and SSA2 members using the partitioned D‐analysis. The results presented here resolve the long running discussion of whether there is mating compatibility between the current highly abundant SSA1‐SG1 and SSA1‐SG2 whitefly populations currently widespread in eastern Africa, and a SSA2 population representing the species responsible for the high populations at the height of the CMD pandemic in EA (Legg *et al*., 2002; Sseruwagi *et al*., 2005).

In contrast to our and Wosula *et al*. (2017) results, Maruthi *et al*. (2004) reported partial reproductive compatibility between SSA2 (UgCas‐Nam) and SSA1‐SG3 (TzCas‐Mtw) *B. tabaci* in an earlier mating study carried out between the two species. The observed partial reproductive compatibility of 27% female progeny in the study of Maruthi *et al*. (2004) between SSA2 and SSA1‐SG3 *B. tabaci* species might have been due to the “virgin” females in the interpopulation crossings possibly having mated prior to the crossing bioassays. Although Maruthi *et al*. (2004) state that virgin females were used by collecting individual female whiteflies within 2 h at the onset of the photophase, studies carried out by Avidov (1956) and Luan *et al*. (2008) showed that copulation could occur within 2 h after emergence in *B. tabaci* species. To avoid the use of already mated females in our study, red‐eyed individual fourth instar nymphs were excised and allowed to emerge in isolation in separate glass tubes before being used in the mating bioassays. This procedure guaranteed that the females used in our mating bioassays were virgins.

The lack of production of female progeny between SSA1‐SG1/‐SG2 and SSA1‐SG3 whiteflies demonstrated mating incompatibility between these whiteflies, thereby supporting our second hypothesis, that two distinct species occur within the SSA1 whiteflies. Phylogenomic relationships revealed through using whole genome SNPs were in agreement with the mating experiments, which both support that SSA1‐SG1 and SSA1‐SG2 have a close and distinct relationship compared to SSA1‐SG3. The congruence in the results obtained by the mating assays and phylogenetic relations is in agreement with earlier reports, which indicated differences in geographical distribution and biological traits for SSA1‐SG1/‐SG2 and SSA1‐SG3 whiteflies. The SSA1‐SG1/SG2 is the whitefly species associated with high populations on cassava in areas around the Lake Victoria Basin (LVB) (Legg *et al*., 2014; Ghosh *et al*., 2015; Tajebe *et al*., 2015), while SSA1‐SG3 is associated with low populations in the coastal Region (CR) of EA and southern Tanzania (Ghosh *et al*., 2015; Wosula *et al*., 2017; Mugerwa *et al*., 2019). Both whitefly species are efficient at transmitting cassava mosaic geminiviruses (CMGs) occurring in their respective localities. Furthermore, a study by Mugerwa *et al*. (2019) showed that there were significant differences in the fecundity and survival rate between SSA1‐SG1 and SSA1‐SG3 whiteflies collected from the LVB and CR of Tanzania, respectively. SSA1‐SG1 whiteflies possessed a higher fecundity and survival rate on both healthy and CMB‐infected cassava plants than the SSA1‐SG3 whiteflies. A combination of the aforementioned biological differences, as well as whole genome SNPs differences observed among SSA1 whiteflies confirms the presence of distinct species within this group.

The phylogenetic relationships among SSA1 subgroups obtained from whole genome SNPs conflicted with those from the full mitogenomes. SSA1‐SG1 and SSA1‐SG2 grouped together but separate from SSA1‐SG3 in the former, while SSA1‐SG3 and SSA1‐SG2 grouped together but separate from SSA1‐SG1 in the latter. Similarly, previous studies have reported inconsistences between phylogenetic topologies generated using mitochondrial and nuclear genome data, indicating the importance of evaluating more gene markers in determining relationships between species (Cameron *et al*., 2004; Spinks & Shaffer, 2009; Wahlberg *et al*., 2009; Wang *et al*., 2017). Our study supports previous studies (Cameron *et al*., 2004; Spinks & Shaffer, 2009; Wahlberg *et al*., 2009; Wang *et al*., 2017) that concluded that the mitochondrial genome data alone is not sufficient to unambiguously resolve the relationships of some organisms. The lack of congruence between relationships generated from mitogenomes versus nuclear genes obtained in our study supports previous studies (Cameron *et al*., 2004; Spinks & Shaffer, 2009; Wahlberg *et al*., 2009; Wang *et al*., 2017) highlighting that inferences based on a single locus/marker, and mtDNA, in particular, should be interpreted with caution. It is therefore important to integrate nuclear genomic studies to existing mtDNA studies for other species within *B. tabaci* complex in order to acquire an improved phylogeny and understanding of relationships among its members.

Production of female progeny between SSA1‐SG1 and SSA1‐SG2 *B. tabaci* populations demonstrated reproductive compatibility under insectary conditions. The viability of F1 female progeny from reciprocal crosses was confirmed, adding further support for SSA1‐SG1 and SSA1‐SG2 representing the same species. In order to demonstrate that no contamination errors occurred while setting up interpopulation crosses 1♀ SSA1‐SG1 × 3♂ SSA1‐SG2 and 1♀ SSA1‐SG2 × 3♂ SSA1‐SG1 and that the hybrids produced were genuine, RFLP analysis was conducted on randomly selected parents and offspring. RFLP results of hybrids and their male siblings were identical to those of their female parent but different from their male parents, as expected with mitochondrial DNA being maternally inherited (Galtier *et al*., 2009).

*Wolbachia*‐induced cytoplasmic incompatibility occurs when the sperm from a *Wolbachia*‐infected male fertilizes an uninfected egg resulting in zygotic death in diploid species or male production in haplodiploid species (Werren, 1997). A reciprocal cross between the sperm from the uninfected male and *Wolbachia*‐infected egg yields viable diploid species (Werren, 1997). Cytoplasmic incompatibility induced by *Wolbachia* was reported in mosquitoes, *Culex pipiens* (Yen & Barr, 1971), alfalfa weevils, *Hypera postica* (Leu *et al*., 1989) and fruit flies, *Drosophila simulans* (Binnington & Hoffmann, 1989). Results presented here show compatibility between *Wolbachia*‐free SSA1‐SG1 and *Wolbachia*‐infected SSA1‐SG2 *B. tabaci* populations in both directions, with a high proportion of female progeny (59.1% ± 6.0%) occurring in a direction where *Wolbachia*‐free SSA1‐SG1 is the female parent. The *Wolbachia* present in SSA1‐SG2 thus does not induce cytoplasmic incompatibility. Partial 16S rDNA sequences of *Wolbachia* detected in SSA1‐SG2 *B. tabaci* showed 100% and 99% nt identity to the partial 16S rDNA sequences of *Wolbachia* detected in *C. pipiens* (accession no. X61768) (O'Neill *et al*., 1992) and *D. mauritiana* (accession no. U17060) (Giordano *et al*., 1995), respectively. These results agree with Ghosh *et al*. (2015) who also identified partial 16S rDNA sequences of *Wolbachia* infecting SSA1‐SG1 and SSA1‐SG2 *B. tabaci*, 100% identical to that infecting mosquitoes. Although the partial 16S rDNA sequences of *Wolbachia* detected in *C. pipiens* and *D. mauritiana* were 99%–100% identical to that infecting SSA1‐SG2 *B. tabaci*, it did not induce cytoplasmic incompatibility in the latter.

Due to the significance of the pest *B. tabaci*, considerable *mtCO1* and “omic” datasets have been published and these include *B. tabaci* draft genome datasets of various members of the species complex (MEAM1, MED, SSA‐ECA, and Asia II 1) (Chen *et al*., 2016, 2019; Xie *et al*., 2017; Hussain *et al*., 2019). The presence of the SSA‐ECA ( = SSA1‐SG1) draft genome, although incomplete, greatly improved the quality and accuracy of SNP calling due to its close relationship to the SSA1 populations used in this study. Similarly, Wosula *et al*. (2017) recently delineated African cassava colonizing whitefly populations into six distinct major groups using 7453 genome‐wide SNPs from NextRAD sequencing mapping to the MEAM1 genome reference. Subsequently, Elfekih *et al*. (2019) found that a species termed SSA4 species should in fact be classified as SSA2 species based on 14 358 genome‐wide SNPs from 63 cassava whitefly individuals. It is clear that different approaches to SNP capture and different sequencing strategies can influence conclusions drawn about the populations/species used. Therefore, it is difficult to tell which sequencing strategy is better in species identification. However, the above studies all provide new insights into the delimitation of the cryptic species using genomic wide SNPs, indicating the need to use multiple loci for more accurate species identification.

## Conclusion

Our study illustrates the importance of comparing genome data with mating compatibility studies to confirm species identity. Nuclear markers were revealed to provide a more accurate representation of species diversity within the *B. tabaci* species complex than the partial mitochondrial sequence markers that are currently used by the *B. tabaci* research community for species delimitation studies (Maruthi *et al*., 2004; Sseruwagi *et al*., 2005; Dinsdale *et al*., 2010; Legg *et al*., 2014; Ghosh *et al*., 2015; Wosula *et al*., 2017; Mugerwa *et al*., 2018; Vyskočilová *et al*., 2018). Although multiple methods (molecular markers and mating experiments) are required to determine species status within *B. tabaci* members, whole genomic‐scale SNPs could be an efficient way to monitor the genetic compositions. In this study, the cost of obtaining enough genomic information from a population using whole genome shotgun approach was approximately $400. The success of this approach on a single whitefly suggests a similar approach would be beneficial to resolve other cryptic insect species complexes of economic importance.

## Authors contributions

Conceptualization, JC, SS, and PS. Methodology, HM, HW, and JC. Formal analysis, HM, HW, SS, JC, and PS. Investigation, HM and HW. Resources, SS and JC. Data curation, HM and HW. Writing—original draft preparation, HM and HW. Writing—review & editing, JC, SS, and PS. Visualization, HM and HW. Supervision, SS, JC, and PS. Project Administration, SS and JC. Funding acquisition, SS and JC.

## Data accessibility

The four mitogenome sequences obtained in this study are deposited in GenBank under the accession numbers MT872660 (SSA2), MT872661 (SSA1‐SG1), MT880240 (SSA1‐SG3), and MT872662 (SSA1‐SG2). The whole‐genome shotgun project has been deposited at GenBank under the SRA accession number PRJNA655126.

## Disclosure

The authors declare that they have no conflict of interest.

## Supporting information


**Table S1** Primer sequences and annealing temperatures for PCR amplification of mtCO1 gene and endosymbiont bacteria.
**Table S2** Comparison of the mean ± SE number of progeny produced by female *Bemisia tabaci* parents pooled from three interpopulation and one intrapopulation crossing experiments.
**Table S3** Mean ± SE number of F2 progeny and percentage of females in the F2 progeny produced by reciprocal crosses between F1 progeny generated from reciprocal crosses of SSA1‐SG1 and SSA1‐SG2 *B. tabaci*.
**Table S4** Endosymbiotic bacteria status in three whiteflies (two females and one male) sampled from each of the four colonies established at NRI. Whitefly colonies were established from individual female and male whiteflies collected from Dar es Salaam, Tanzania; Kayingo and Kiboga, Uganda. Sub‐Saharan Africa 1 (SSA1) composed of subgroup (SG) 1–3 while SSA2 represent Sub‐Saharan Africa 2 species.
**Table S5** Pairwise comparison of the partial Wolbachia 16S ribosomal DNA (728 bp), expressed as percentage identity matrix between adult *Bemisia tabaci*, *Drosophila mauritiana* and *Culex pipens*, calculated in Clustal Omega (Sievers *et al*., 2014) available at EBI website. The *D. mauritiana* and *C. pipens* Wolbachia 16S ribosomal DNA sequences were obtained from GenBank. Individual *B. tabaci* was collected from SSA1‐SG2 colony established from Kayingo, Uganda.
