# Supplementary material for: Whole‐genome single nucleotide polymorphism and mating compatibility studies reveal the presence of distinct species in sub‐Saharan Africa Bemisia tabaci whiteflies
Source: Insect Sci. 2020 Nov 30;28(6):1553–66. doi: 10.1111/1744-7917.12881 (PMC9292209; doi:10.1111/1744-7917.12881)
Supplement: Supplementary file 1 — Table S1 Primer sequences and annealing temperatures for PCR amplification of mtCO1 gene and endosymbiont bacteria. Table S2 Comparison of the mean ± SE number of progeny produced by female Bemisia tabaci parents pooled from three interpopulation and one intrapopulation crossing experiments. Table S3 Mean ± SE number of F2 progeny and percentage of females in the F2 progeny produced by reciprocal crosses between F1 progeny generated from reciprocal crosses of SSA1‐SG1 and SSA1‐SG2 B. tabaci. Table S4 Endosymbiotic bacteria status in three whiteflies (two females and one male) sampled from each of the four colonies established at NRI. Whitefly colonies were established from individual female and male whiteflies collected from Dar es Salaam, Tanzania; Kayingo and Kiboga, Uganda. Sub‐Saharan Africa 1 (SSA1) composed of subgroup (SG) 1–3 while SSA2 represent Sub‐Saharan Africa 2 species. Table S5 Pairwise comparison of the partial Wolbachia 16S ribosomal DNA (728 bp), expressed as percentage identity matrix between adult Bemisia tabaci, Drosophila mauritiana and Culex pipens, calculated in Clustal Omega (Sievers et al., 2014) available at EBI website. The D. mauritiana and C. pipens Wolbachia 16S ribosomal DNA sequences were obtained from GenBank. Individual B. tabaci was collected from SSA1‐SG2 colony established from Kayingo, Uganda. [file INS-28-1553-s001.docx]

**Table S1** Primer sequences and annealing temperatures for PCR amplification of *mtCO1* gene and endosymbiont bacteria.

| **Target gene** | **Primer name and Sequence (5’→3’)** | **PCR program for 35 cycles** | **Reference** | **Amplicon length** |
| --- | --- | --- | --- | --- |
| *mtCO1* | 2195Bt (5′-TGRTTTTTTGGTCATCCRGAAGT-3′)  C012/Bt-sh2 (5′-TTTACTGCACTTTCTGCC-3′) | 94°C, 30 s  52°C, 30 s  72°C, 1 min | Mugerwa *et al*., 2018 | 864 |
| *Portiera* 16S rDNA | 28F (TGCAAGTCGAGCGGCATCAT)  1098R (AAAGTTCCCGCCTTATGCGT) | 94°C, 30 s  58°C, 30 s  72°, 1 min | Zchori-Fein and Brown, 2002 | 1050 bp |
| *Arsenophonus* 23S rDNA | Ars23S-1 (CGTTTGATGAATTCATAGTCAAA)  Ars23S-2 (GGTCCTCCAGTTAGTGTTACCCAAC) | 94°C, 30 s  58°C, 30 s  72°C, 1 min | Chiel *et al.*, 2007 | 750 bp |
| *Cardinium* 16S rDNA | Card-F (TAGACACACACGAAAGTTCATGT)  Card-R (GCATGCAATCTACTTTACACTGG) | 94°C, 30 s  57°C, 30 s  72°C, 1 min | Ghosh *et al*., 2015 | 650 bp |
| *Hamiltonella* 16S rDNA | Hb-F (TGAGTAAAGTCTGGGAATCTGG)  Hb-R (AGTTCAAGACCGCAACCTC) | 94°C, 30 s  58°C, 30 s  72°C, 1 min | Gueguen *et al*., 2010 | 730 bp |
| *Fritschea* 23S rDNA | Frit-F (GAGTTTGATCATGGCTCAGATTG)  Frit-R (GCTCGCGTACCACTTTAAATGGCG) | 94°C, 30 s  62°C, 30 s  72°C, 1 min | Gueguen *et al*., 2010 | 630 bp |
| *Rickettsia* 16S rDNA | Rb-F (GCTCAGAACGAACGCTATC)  Rb-R (GAAGGAAAGCATCTCTGC) | 94°C, 30 s  58°C, 30 s  72°C, 1 min | Gottlieb *et al*., 2006 | ~900 bp |
| *Wolbachia* 16S rDNA | Wol16S-F (CGGGGGAAAAATTTATTGCT)  Wol16S-R (CCCCATCCCTTCGAATAGGTAT) | 94°C, 30 s  58°C, 30 s  72°C, 1 min | Heddi *et al*., 1999  Ghosh *et al*., 2015 | 730 bp |

**Table S2** Comparison of the mean ± SE number of progeny produced by female *Bemisia tabaci* parents pooled from three inter-population and one intra-population crossing experiments.

| Identity of female parent | No. of replicates | Mean no. of progeny ± SE |
| --- | --- | --- |
| SSA1-SG1 | 28 | 30.7±3.2a |
| SSA1-SG2 | 29 | 43.1±4.3ab |
| SSA1-SG3 | 35 | 36.4±3.3ab |
| SSA2 | 37 | 44.9±3.9b |

Means followed by the different letters differ significantly at *P*<0.05.

**Table S3** Mean ± SE number of F2 progeny and percentage of females in the F2 progeny produced by reciprocal crosses between F1 progeny generated from reciprocal crosses of SSA1-SG1 and SSA1-SG2 *B. tabaci*.

| Crosses  1♀ × 3♂ | No. of replicates | Mean no. of progeny ± SE | % female progeny |
| --- | --- | --- | --- |
| F1 of (♀ SSA1-SG1 **×** ♂ SSA1-SG2) | 6 | 87.8±12.1 | 48.0±11.3 |
| F1 of (♀ SSA1-SG2 **×** ♂ SSA1-SG1) | 6 | 43.4±6.1 | 25.9±14.3 |

*Bemisia tabaci* used in these crosses were collected from Kayinyo, Uganda.

**Table S4** Endosymbiotic bacteria status in three whiteflies (two females and one male) sampled from each of the four colonies established at NRI. Whitefly colonies were established from individual female and male whiteflies collected from Dar es Salaam, Tanzania; Kayingo and Kiboga, Uganda. Sub-Saharan Africa 1 (SSA1) composed of subgroup (SG) 1–3 while SSA2 represent Sub-Saharan Africa 2 species.

| **Population/Endosymbiont** | ***Portiera*** | ***Arsenophonus*** | ***Cardinium*** | ***Fritschea*** | ***Hamiltonella*** | ***Rickettsia*** | ***Wolbachia*** |
| --- | --- | --- | --- | --- | --- | --- | --- |
| SSA1-SG1 | 3/3 | 0/3 | 0/3 | 0/3 | 0/3 | 0/3 | 0/3 |
| SSA1-SG2 | 3/3 | 0/3 | 0/3 | 0/3 | 0/3 | 0/3 | 3/3 |
| SSA1-SG3 | 3/3 | 0/3 | 0/3 | 0/3 | 0/3 | 0/3 | 0/3 |
| SSA2 | 3/3 | 0/3 | 0/3 | 0/3 | 0/3 | 0/3 | 0/3 |

**Table S5** Pairwise comparison of the partial *Wolbachia* 16S ribosomal DNA (728 bp), expressed as percentage identity matrix between adult *Bemisia tabaci*, *Drosophila mauritiana* and *Culex pipens*, calculated in Clustal Omega (Sievers *et al.* 2014) available at EBI website. The *D. mauritiana* and *C. pipens* *Wolbachia* 16S ribosomal DNA sequences were obtained from GenBank. Individual *B. tabaci* was collected from SSA1-SG2 colony established from Kayingo, Uganda.

|  | Insect species | 1 | 2 | 3 |
| --- | --- | --- | --- | --- |
| 1 | *Drosophila mauritiana* U17060 | - |  |  |
| 2 | *Bemisia tabaci* SSA1-SG2 18 Kayingo colony | 99.2 | - |  |
| 3 | *Culex pipens* X61768 | 99.2 | 100.0 | - |
